# Ligature of a Polypus of the Bladder

**Published:** 1845-04

**Authors:** 


*Ligature of a Polypus of the Bladder.*—Augusta S., 45 years of age, unmarried, had long complained of pain and difficulty of making water, of bloody urine, &c.; when one day suddenly a dark red body, which bled freely, presented itself between the labia. Careful examination discovered it to be a polypus, the size of a hen's egg, and projecting from the urethra, which could readily be entered with two fingers, and the pedicle of the polypus, which was one-third of an inch thick, followed into the bladder, without however getting to its point of attachment. I succeeded in throwing a ligature round it by means of two elastic catheters, and then with a proper canula, constricted it as far within the bladder as I could reach. On the third day the ligature was tightened, and on the 6th the polypus fell off. The patient recovered completely.—*Dr. Thiénemann, Medicinishe Zeitung.*

